# Bone morphogenetic protein signaling in vertebrate motor neurons and neuromuscular communication

**DOI:** 10.3389/fncel.2014.00453

**Published:** 2015-01-27

**Authors:** Nelson Osses, Juan P. Henríquez

**Affiliations:** ^1^BMP Research Group, Institute of Chemistry, Faculty of Sciences, Pontificia Universidad Católica de ValparaísoValparaíso, Chile; ^2^Laboratory of Developmental Neurobiology, Department of Cell Biology, Faculty of Biological Sciences, Millennium Nucleus of Regenerative Biology, Center for Advanced Microscopy (CMA Bio-Bio), Universidad de ConcepciónConcepción, Chile

**Keywords:** BMP, motor neuron, muscle, NMJ, synapse

## Abstract

An accurate communication between motor neurons and skeletal muscle fibers is required for the proper assembly, growth and maintenance of neuromuscular junctions (NMJs). Several signaling and extracellular matrix molecules play stimulatory and inhibitory roles on the assembly of functional synapses. Studies in Drosophila have revealed crucial functions for early morphogens, such as members of the Wnt and Bone Morphogenetic Proteins (BMP) signaling pathways, during the assembly and maturation of the NMJ. Here, we bring together recent findings that led us to propose that BMPs also work in vertebrate organisms as diffusible cues to communicate motor neurons and skeletal muscles.

## Introduction

The establishment of functional neuromuscular synapses is essential for the coordinated movement of the organisms. The accurate communication between motor neurons and skeletal muscle fibers is crucial for proper synaptic assembly, growth and maintenance. In this regard, it has been demonstrated that the embryonic formation of the vertebrate neuromuscular junction (NMJ) is driven by signaling and extracellular matrix molecules derived from presynaptic motor neurons and from postsynaptic muscle cells that are intermingled to assemble functional synapses. For instance, the early assembly of postsynaptic densities, characterized by the clustering of acetylcholine receptors (AChRs) on the surface of developing muscle cells, does not require neuronal inputs (Flanagan-Steet et al., 2005; Lin et al., 2008; Jing et al., 2009). However, neuronal cues refine this postsynaptic pattern by, in one hand, eliminating AChRs from extrasynaptic regions via a neurotransmitter-dependent mechanism and, simultaneously, by maintaining and maturing postsynaptic densities in neuromuscular contacts through the action of Agrin (Lin et al., 2005; Misgeld et al., 2005; An et al., 2010). The MuSK activator Agrin has a separate role essential for postnatal maintenance of neuromuscular synapses (Tezuka et al., 2014). Also, presynaptic differentiation is controlled by the coordinated action of extracellular molecules (Nishimune et al., 2004; Fox et al., 2007; Carlson et al., 2010). For instance, members of the fibroblast growth factor family are required for the initiation of presynaptic terminals, laminins act as crucial molecules for presynaptic maturation, and collagens positively affect the maintainance of proper NMJs (Fox et al., 2007). Therefore, multiple signaling pathways are interplayed to orchestrate the correct differentiation and positioning of functional NMJs.

An emerging concept in the field is the participation of signaling molecules of the early development on the wiring of synaptic contacts, including the NMJ. These molecules, named morphogens, have the ability to induce distinct cellular responses in a concentration-dependent manner. Major players of this effect are ligands of the Wnt pathways (for reviews, see Henríquez and Salinas, 2012; Koles and Budnik, 2012; Park and Shen, 2012). Different Wnt pathways are able to induce the early aneural assembly of postsynaptic densities by binding the Agrin-interacting protein MuSK (Jing et al., 2009), and, at the same time, they also regulate the formation of well positioned NMJs, possibly acting through their cognate Frizzled receptors (Packard et al., 2002; Mathew et al., 2005). Growing evidence, mainly obtained from *Drosophila*, reveals that pathways activated by members of the bone-morphogenetic proteins (BMP), a family of secreted ligands belonging to the transforming growth factor-β (TGF-β) superfamily of signaling proteins, also play key roles on neuromuscular synaptogenesis (Marqués, 2005). The role of TGF-β/BMP pathways in invertebrate and vertebrate motor neurons has been reviewed elsewhere (Katsuno et al., 2011). In this review, we focus in highlighting recent insights into the possibility that BMPs also work in vertebrate organisms as diffusible signals to ensure proper communication between motor neurons and skeletal muscles.

## Signaling pathways activated by BMP ligands

Based on their high sequence identity, the BMP family members (>20) have been classified in multiple subgroups that are conserved between vertebrate and invertebrate species (Kishigami and Mishina, 2005). Even though BMPs were first named based on their ability to induce ectopic bone formation (Urist, 1965; Wozney et al., 1988), different studies have shown that they are multifunctional proteins affecting a diversity of biological responses. These include early developmental processes, such as dorso-ventral patterning, patterning of the body axes, early patterning of the central nervous system, as well as the specification of several tissues and organs (Zhao, 2003; De Robertis and Kuroda, 2004; Kishigami and Mishina, 2005; Sieber et al., 2009).

**Figure 1:**
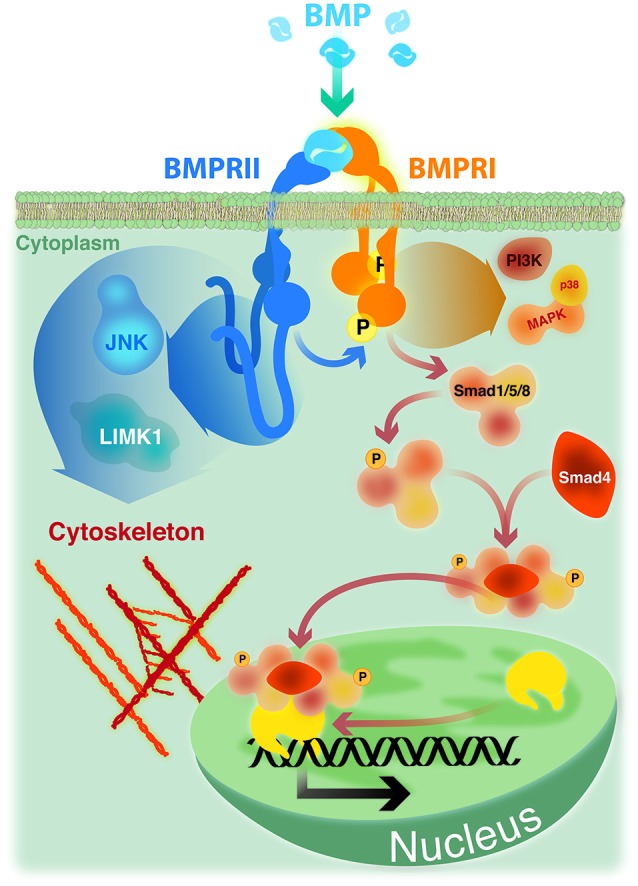
**BMP signaling**. Smad signaling is initiated upon phosphorylation of Smad-1, -5 or -8 by a heteromeric complex of BMPRII, BMPRI and BMP ligand. Phosphorylated Smads recruit Smad-4 and translocate to the nucleus where, in association with co-repressors or co-activators, regulate the transcription of specific target genes. Non Smad pathways, such as p38 MAP kinase and PI3 kinase, are also initiated by heteromeric complexes. The activity of cytoskeleton regulators mediating actin remodeling (LIMK1) and microtubule stabilization (JNK) are dependent on its ability to bind to the long cytoplasmic tail of BMPRII.

Early events in BMP signaling involve the formation of heteromeric complexes of two types of transmembrane receptors with serine/threonine kinase activities, named type I and type II. Receptors of both types are needed to form a functional complex for signal transduction (Figure 1; Yamashita et al., 1994; Liu et al., 1995). BMPs can interact with three distinct type I (ActRIA, BMPRIA and BMPRIB) or type II receptors (BMPRII, ActRIIA and ActRIIB). This interaction relies on their affinity, but also on the specific expression pattern of the different BMP receptors (Sebald et al., 2004; Lin et al., 2006). Detailed analyses of BMP pathways came from studies of cell responses to BMP-2 (Sieber et al., 2009). BMP-2 binding to a preformed heteromeric complex of BMPRII and BMPRI initiate a classical Smad-dependent signaling pathway (see Figure 1; Gilboa et al., 2000; Nohe et al., 2002). Upon BMP-2 binding, BMPRI is phosphorylated by BMPRII. Activated BMPRI initiates the phosphorylation of specific receptor-regulated Smad proteins, namely R-Smad-1, -5 or -8, which form heteromeric complexes with the common mediator Smad-4. Such complexes translocate to the nucleus to regulate the transcription of specific target genes in cooperation with co-repressors or co-activators (Shi and Massagué, 2003; Nohe et al., 2004; Miyazono et al., 2005).

In addition, BMP-2 is able to induce non-Smad pathways, such as activation of p38 mitogen-activated protein kinase (MAPK) (Nohe et al., 2004; Figure 1). Activation of p38 MAPK is the consequence of BMP-2 binding to its high affinity receptor BMPRI which induce the recruitment of BMPRII, thus leading to the formation of the signaling heteromeric complex (Gilboa et al., 2000; Nohe et al., 2002). Also, BMPs have the ability to trigger other Smad-independent pathways, which are mediated by different intracellular mediators, including ERK, the nuclear factor kappa beta (NFkB), and phosphoinositide 3-kinase (PI3 Kinase; Sieber et al., 2009; Bragdon et al., 2011). The activation of different BMP signaling pathways defines their wide ranges of different biological effects. These signaling cascades are finely regulated by the temporospatial expression of regulatory factors, such as antagonists that bind and inactivate the ligands, co-receptors of the cell surface having stimulatory or inhibitory roles, intracellular regulatory proteins, and specific co-repressors or co-activators that regulate the transcription of specific target genes (Miyazono et al., 2005; Sieber et al., 2009).

BMPs have a very dynamic role in the nervous system including their ability to regulate early steps of neuralization to patterning, neural stem cell proliferation, selfrenewal, lineage specification and neuronal function (dendritic and axonal growth as well as synapse formation and stabilization) by either Smad-dependent or Smad-independent signaling (see Gámez et al., 2013, Front. Cell. Neurosci.; this issue).

## BMPRII role in neuronal outcome

All receptors from the TGF-β superfamily are arranged in three conserved moieties: an extracellular ligand binding domain, a transmembrane segment, and an intracellular serine/threonine kinase domain (Sebald et al., 2004). Among receptors of the TGF- β superfamily, an outstanding feature of BMPRII is the presence of a long Carboxy terminal tail following its kinase domain, which corresponds to ~500 amino acids, representing the 50% of the protein (Waite and Eng, 2003). The long C-terminal tail of BMPRII is also present in its corresponding homologs in various vertebrate and invertebrate species (Estevez et al., 1993; Ishikawa et al., 1995; Aberle et al., 2002).

An essential role of BMP signaling linked to synaptic growth, synapse stability, and homeostatic plasticity has been shown in Drosophila. Remarkably, these studies have demonstrated that a key component of BMP signaling affecting the NMJ is wishful thinking (Wit), the BMPRII homolog. Wit mutant larvae show a significant impairment of the morphology and function of the neuromuscular synapse (Aberle et al., 2002; Marqués et al., 2002). The expression of Wit in these mutants rescued the normal phenotype, demonstrating a key role for the signaling mediated by this receptor on the behavior of presynaptic neurons at the invertebrate NMJ (Aberle et al., 2002; Marqués et al., 2002). Impaired motor neuron synaptic growth and function has also been demonstrated in loss of function mutants either for the BMP-7 homolog Glass bottom boat (Gbb), the BMPRI Saxophone (Sax) or the Smad homologs Mad and Medea (McCabe et al., 2003; Rawson et al., 2003; Eaton and Davis, 2005; McCann et al., 2005). Gbb is expressed in presynaptic neurons and postsynaptic muscle (James and Broihier, 2011). A role of Gbb and Wit in the expression of presynaptic proteins that are required for synaptic homeostasis independent of BMP-dependent regulation of synaptic growth or stability has been proposed (Goold and Davis, 2007), whereas muscle-derived Gbb is involved in retrograde signaling related to synaptic growth (James and Broihier, 2011) and synapse maturation (Berke et al., 2013). Hence, in invertebrates Wit is required in cell soma of motor neurons as well as for retrograde signaling to activate canonical Smad-dependent BMP signaling that works through transcriptional mechanism to control motor neuron behavior.

Additionally, a role of BMP signaling in synaptic stability has been shown. In this regard, mutants of Wit receptor showed a strong increase of synaptic footprints (regions of the NMJ where the terminal nerve once resided and has retracted) as compared to those observed in mutants of other canonical BMP signaling molecules. A potential mechanism accounting for this effect is related to the ability of the C-terminal domain of Wit to interact with the actin cytoskeleton modulator LIM Kinase1 (DLIMK1) in motor neurons to stabilize the NMJ. Remarkably, this interaction is not required for Smad-mediated synaptic growth (Eaton and Davis, 2005). In line with this observation, rapidly generated presynaptic varicosities, referred as ghost boutons, require retrograde Gbb signal and are locally regulated by Wit that, through an interaction with LIMK, regulate the synaptic actin cytoskeleton and inhibits bouton budding (Piccioli and Littleton, 2014). Therefore, Smad-dependent and independent signaling pathways via the Wit receptor expressed by invertebrate motor neurons are involved in synaptic outcomes at the NMJ. Supporting a central role of BMPRII in vertebrate species, it has been shown that mice spinal motor neurons expressed detectable mRNA levels of all type I and type II receptors, but BMPRII is at least 26 times more abundant than the other receptors (Wang et al., 2007a).

Different studies have shown that Smad-independent BMP induced pathways are related to local signals that involve cytoskeleton arrangements. In this regard, BMP-mediated cell migration, axon and dendrite growth, or axon guidance require activation of the small GTPase Cdc42, PI3-K, p38 MAPK, c-Jun N-terminal kinase (JNKs) or LIMK (Foletta et al., 2003; Lee-Hoeflich et al., 2004; Eaton and Davis, 2005; Wen et al., 2007; Gamell et al., 2008, 2011; Podkowa et al., 2010; Hiepen et al., 2014). Interestingly, BMPRII has been involved in most of the studies localizing or coordinating this local signaling. For instance, upon BMP-2 stimulation, the p38 downstream effector Hsp25, that regulates cytoskeletal dynamics, colocalizes with BMPRII (Gamell et al., 2011). In addition, BMP-2 induced planar cell polarity and actin dependent lamellipodia formation mediated by PI3-K involve the binding of the PI3K regulatory subunit p55γ to BMPRII, irrespective of the presence of the C-terminal tail (Hiepen et al., 2014). Also, BMPRII lacking the C-terminal tail is able to activate Smad pathways in response to BMP-2 (Nohe et al., 2002), suggesting that this domain of BMPRII could play other regulatory roles.

The first described function of BMPRII C-terminal tail was the demonstration that the activity of LIM kinase 1 (LIMK1), a regulator of actin dynamics, was reduced by its binding to this terminal region of the BMPRII (Foletta et al., 2003). Afterwards, it was showed that the positive effect of BMP-7 on dendritogenesis of mouse cortical neurons required the association of LIMK1 with the BMPRII C-terminal tail, which synergized with the Rho GTPase Cdc42 to activate the LIMK1 catalytic activity (Lee-Hoeflich et al., 2004). Similarly, the binding of JNK proteins to the C-terminal tail of BMPRII was also required both for microtubule stabilization and for the BMP-7 induced dendritogenesis in primary cortical neurons (Podkowa et al., 2010). BMP-7 also induces an attractive response of spinal neuron axons by eliciting asymmetric actin polymerization/stabilization through the spatial regulation of ADF/cofilin activity via interaction of LIMK1 with the BMPRII C-terminal tail (Wen et al., 2007). Hence, BMP induced Smad-independent signals elicited by type I receptors may have cytoskeleton effects depending on the BMPRII relationship with adaptor proteins (Figure 1).

In the following sections, we will summarize recent findings revealing that signaling pathways activated by BMP ligands could regulate the behavior of two of the main constituents of the neuromuscular synapse in vertebrate species, muscle cells and motor neurons, and propose a model of how these BMP-mediated effects could combine at the neuromuscular connection.

## BMP signaling in skeletal muscle

**Figure 2:**
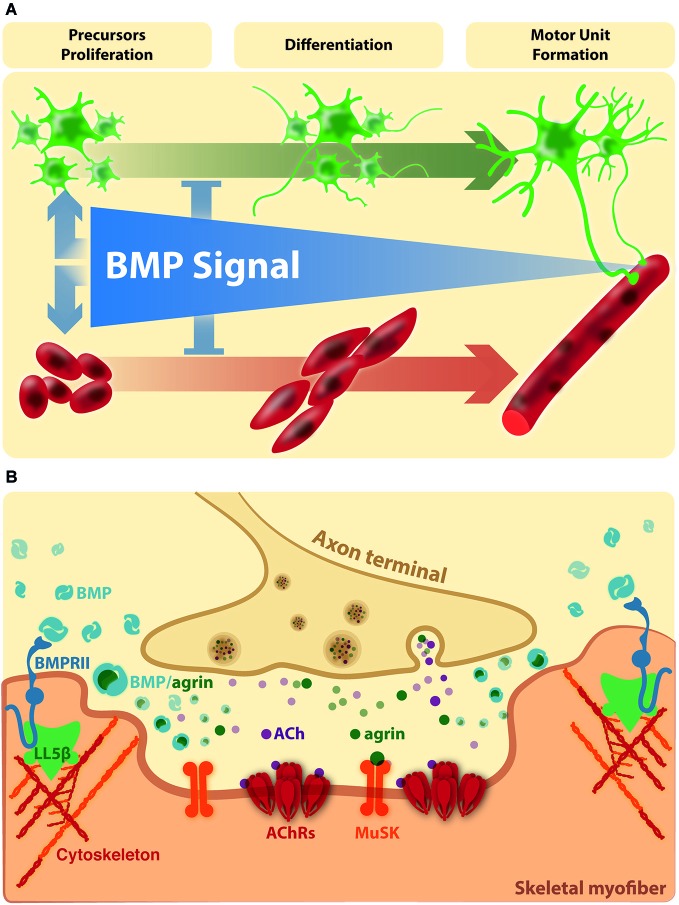
**BMP signaling on the connectivity of the vertebrate neuromuscular synapse. (A)** In vertebrates, the evidence suggests that BMPs stimulate the amplification of muscle and motor neurons precursors and repress precocious differentiation. At this stage, the BMP dependent effects are mainly Smad dependent. At later stages, BMP signaling becomes restricted to the site of innervation. **(B)** Here, activation of BMP pathways could be involved in NMJ formation, maturation and/or maintenance. Agrin and BMPs could modulate the extracellular distribution and availability of each other for receptor binding in synaptic domains. In turn, local BMP-dependent pathways could affect cortical actin rearrangements at extrasynaptic domains (see text for details).

During embryonic development, skeletal muscles derive from mesodermal precursor cells by successive steps. The first event involves specification of precursor cells to the myogenic lineage. These committed proliferating cells, named myoblasts, withdraw from the cell cycle and begin to express muscle-specific genes. Finally, differentiating myoblasts fuse to generate multinucleated muscle fibers. During the process, a subpopulation of myoblasts does not differentiate and remains associated to the fiber as quiescent satellite cells (Chargé and Rudnicki, 2004). After myofiber damage, satellite cells are activated to generate committed myoblasts that proliferate and differentiate to repair damaged fibers and generate new multinucleated fibers (Chargé and Rudnicki, 2004). *In vivo* models of damage-induced muscle regeneration show the activation of pSmad1/5/8 in Tibialis anterior muscle lysates after 1 day of injury, which is still visible after 3 days, and became undetectable at day seven, suggesting that activated satellite cells express pSmad1/5/8 (Clever et al., 2010). In agreement, pSmad1/5/8 is detected in satellite cells and myoblasts after 3 days in models of regenerating Gastrocnemius or Tibialis anterior muscle of adult mice (Clever et al., 2010; Ono et al., 2011). These findings suggest that the induction of Smad-dependent BMP signaling is a generalized response to muscle injury and seems to be related to activation and expansion of myogenic precursor cells. Accordingly, isolated myofibres with satellite cells show a strong nuclear immunostaining of phosphorylated Smad1/5/8 in activated and proliferating, but not quiescent, satellite cells (Ono et al., 2011). Importantly, in satellite cells induced to differentiate, BMP-4 causes an increase in total cell number of committed cells but a significant fall in the proportion of differentiating cells and their fusion into myotubes. Consistently, blocking the interaction of BMP-4 with its receptors, as well as down-regulation of the BMPRIA or inhibiting the intracellular BMP-Smad signal, induces a faster differentiation (Ono et al., 2011). The aforementioned studies indicate that the BMP pathway inhibits muscle differentiation, but also has the ability to stimulate satellite cell proliferation (Figure 2A). Accordingly, it has been proposed that BMP signaling is important to stimulate the amplification of committed myoblasts and to prevent precocious differentiation during muscle regeneration (Ono et al., 2011).

New insights are also emerging related to the role of BMP signaling in skeletal muscle. Recent findings have demonstrated the role of BMP Smad-dependent signaling in the control of muscle mass, by promoting hypertrophy and counteracting atrophy (Sartori et al., 2013; Winbanks et al., 2013). Gdf6 (encoding BMP13) and Gdf5 (encoding BMP14) are induced in mouse skeletal muscle subjected to denervation, used as a model of muscle atrophy (Sartori et al., 2013; Winbanks et al., 2013). Accordingly, an autocrine signal is proposed as responsible of the increased Smad1/5/8 phosphorylation in muscle that is essential to limit atrophy in denervated muscles (Sartori et al., 2013; Winbanks et al., 2013). Importantly, several BMP genes and BMP receptors are expressed in innervated muscles (Sartori et al., 2013; Winbanks et al., 2013) suggesting that BMP induced signaling is regulated in adult muscle by a mechanism dependent on motor nerve activity. In agreement, phosphorylation of Smad1/5 declined markedly from 1 week after birth until 6 months in mouse skeletal muscle (Winbanks et al., 2013), indicating that BMP dependent signaling in muscle cells is repressed during postnatal maturation.

Agrin, a main motor neuron-derived postsynaptic organizer (Bowe and Fallon, 1995), binds BMP-2 and -4 and decreases their rate of association to the extracellular domain of BMPRIA, thereby inhibiting BMP-induced signaling (Bányai et al., 2010). Thus, it is possible that Agrin plays a role in the control of BMP activity in muscle fibers, by modulating the extracellular distribution and availability of BMPs for receptor binding. Consistent with this idea, BMP-4 has been immunodetected in Soleus muscle fibers, localized in close vicinity to postsynaptic densities at the NMJ (Chou et al., 2013). Indeed, denervated Soleus muscle loose BMP-4 immunoreactivity, reinforcing the idea that this specific localization of BMP-4 in muscle fibers is regulated by a factor derived from motor neurons (Chou et al., 2013). Interestingly, BMP-4 surrounds the postsynaptic density at the NMJ neurons (Chou et al., 2013). This localization of BMP-4 could be the consequence of a local increase of BMPRII, which becomes associated to protein adaptors, such as LIMK1. It is interesting to note that peripheral regions of postsynaptic densities at the NMJ are characterized by the absence of AChR clusters but also by the accumulation of polymerized F-actin; indeed, the actin binding protein cofilin is likely to play a crucial role on the active disaggregation of AChR clusters (Lee et al., 2009). Also, these regions contain the phosphoinositide-binding protein LL5β, which promotes postsynaptic maturation at the NMJ by delimiting AChR-rich areas (Kishi et al., 2005). Therefore, it is tempting to speculate that the specific localization of BMP-4 at the border of postsynaptic densities of the NMJ could have a dual effect on muscle fibers: on one hand, it may represent a sequestering of this BMP ligand to preclude activation of BMP signaling in innervated muscles. On the other hand, it may activate local BMP-dependent pathways involved in the disassembly of AChR clusters at the NMJ (Figure 2B). Remarkably, this notion is supported by recent evidence showing that the BMP-2 induced chemotaxis of mesenchymal progenitor cells depends on the effect of LL5β, likely mediated by the BMPRII, on cortical actin rearrangements (Hiepen et al., 2014).

Collectively, information from models of muscle induced regeneration and neurogenic atrophy lead us to propose that BMP signaling plays local roles on skeletal muscle that are like dependent on motor neuron activity. Probably, the source of BMP is the skeletal muscle; indeed, BMP-4 is expressed in myogenic cells (Kumar et al., 2009; Clever et al., 2010; Chou et al., 2013). Furthermore, as mentioned before, several BMP genes and BMP pathway components are expressed in adult muscle, and the transcript expression levels of some BMPs are induced in skeletal muscle after denervation (Sartori et al., 2013; Winbanks et al., 2013). In addition, Agrin increases mRNA expression and the immunoreactivity of BMP-4 in differentiated C2C12 muscle cells (Chou et al., 2013). Thus, BMPs expressed by muscle cells could play autocrine effects but, considering the contiguous relationship between skeletal muscle and motor neurons, they could also affect motor neuron behavior. This idea is consistent with the aforementioned data pointing to the crucial role that the retrograde secretion of the BMP ligand Gbb plays in the proper nerve terminal development and establishment of the NMJ in Drosophila.

## BMP signaling in motor neurons

In the adult neuronal tissue, BMPs regulate several features of cell behavior. For instance, dendritogenesis, number of neurites, length of neurites and branch points have been shown to be stimulated or inhibited by different BMPs in diverse neuronal types, including cultured sympathetic, cerebral cortical, hippocampal, postnatal cerebellar and peripheral neurons (Iwasaki et al., 1999; Gratacòs et al., 2001; Horbinski et al., 2002; Yabe et al., 2002; Lee-Hoeflich et al., 2004; Matsuura et al., 2007; Anitha et al., 2010).

In motor neurons of vertebrates, a distinctive role of BMPs has not yet been described. However, some evidence came from experimental models of injury. For instance, BMP-2 mRNA is expressed in motor neurons after crush injury of the facial nerve in rabbits (Wang et al., 2007b). Similarly, traumatic injury of the rat spinal cord results in a remarkable up-regulation of BMP-7 and BMP-2 around the injury site during recovery (Setoguchi et al., 2001; Matsuura et al., 2008). It has also been considered that some BMPs induce astroglial differentiation and, according to this, the manipulation of BMP signaling at the injured spinal cord has been mainly intended to evaluate possible functional recovery (Sahni et al., 2010). In this regard, enhanced locomotor activity and axonal regrowth is observed when BMP binding to their receptors is inhibited by administration of noggin—a high affinity soluble antagonist of BMP-2—into the injured spinal cord (Matsuura et al., 2008). Accordingly, a partial functional recovery has been shown after transplantation of neural progenitor cells modified to express noggin into injured mouse spinal cords (Setoguchi et al., 2004). Therefore, from these studies it is possible to propose that BMP signaling opposes to the differentiation of motor neurons. Indeed, BMP-2 plays a negative role on the neurite outgrowth of the motor neuron cell line NSC34 (Benavente et al., 2012). This BMP-2 dependent inhibition of morphological differentiation is accompanied by increased phosphorylation of Smad1/5/8, as well as by an increase in the expression and activity of the Smad-dependent early responsive gene Id1, a negative regulator of the differentiation of neurogenic precursors (Ying et al., 2003; Viñals et al., 2004). Simultaneously, the levels of the neurogenic factor Mash1 are down-regulated by BMP-2 (Benavente et al., 2012). Together, these findings reveal that BMP signaling activation acts as a negative regulator of motor neuron differentiation.

Models of motor neuronal pathologies have also been useful to decipher a potential role of BMP signaling in these neurons. Amyotrophic lateral sclerosis (ALS) is a late-onset neurodegenerative disease characterized by the selective loss of motor neurons, leading to paralysis and death (Pasinelli and Brown, 2006). In familial cases of ALS, 20% corresponds to point mutations of superoxide dismutase-1 (SOD1; Rosen et al., 1993). Indeed, the G93A mutation of SOD1 has been widely used to generate model systems of ALS, either animals or *in vitro*, as they mimic the main clinical, pathological and cellular features of the disease (Gurney et al., 1994; Arciello et al., 2011; DuVal et al., 2014). We have analyzed BMP signaling in the context of ALS by using motor neuron-like NSC34 cells stably expressing wild-type or G93A mutated forms of human SOD1. In undifferentiated cells, phospho Smad 1/5/8 and Id1 levels are significantly higher in NSC34 cells expressing hSODG93A compared to controls, and then they are similarly down-regulated during the differentiation of both cell types (Pinto et al., 2013). Consistently, the basal phospho Smad-dependent transcription of Id1 is 2-fold higher in cells with constitutive expression of hSOD1G93A, as compared to those expressing hSOD1WT, whereas this activity is strongly diminished after 24 and 48 h of differentiation in both cell types (Pinto et al., 2013). As Smad signaling varies inversely with motor neuron differentiation, the impaired morphological differentiation observed in the ALS model may be correlated to up-regulated Smad signaling. It is appealing to note that BMP signaling inhibits the differentiation of precursor motor neuron cells, similarly to what was observed in muscle precursor cells. Comparable to the role of BMP signaling in muscle cells, it has been shown that, during mouse neural development, Smad signaling induced by BMP-2 is required to prevent premature differentiation (Di-Gregorio et al., 2007).

Hereditary Spastic Paraplegia (HSP) is a group of genetic disorders characterized by retrograde axonal degeneration of the corticospinal tract and the posterior columns in the spinal cord that result in progressive spasticity and weakness of the lower limbs (Salinas et al., 2008). Different mutated genes have been identified in HSPs (Dion et al., 2009). Atlastin, spastin, spartin and NIPA1 are among the mutated proteins related to HSP accounting for the majority of clinical cases. Remarkably, common features of these proteins in vertebrates are their localization in endosomal membrane traffic compartments and their ability to influence BMP signaling (Tsang et al., 2009; Fassier et al., 2010). For instance, morpholino-dependent knockdown of the ortholog of human atlastin (*atl1*) in developing zebrafish induced a prominent loss of larvae motility, associated with abnormal axon pathfinding and multiple aberrant branching of spinal motor neurons (Fassier et al., 2010). Interestingly, primary cultures of zebrafish spinal neurons from *atl1* morphants show a significant increase in the amount of nuclear pSmad1/5/8, suggesting that atlastin represses BMP signaling. Consistently, atl1 knockdown defects were rescued by genetic or pharmacologic inhibition of the BMP pathway (Fassier et al., 2010). As for atlastin, enhanced phosphorylation of Smad1/5 has been observed after siRNA-mediated silencing of NIPA1, spastin and spartin in mammalian cells (Tsang et al., 2009). Together, these data support the notion that different endosomal proteins related to HSP act as repressors of BMP Smad-mediated signaling. Thus, deregulations on the trafficking of BMP receptors, having as a consequence enhanced Smad signaling, may produce negative effects on motor neuron behavior or even axonal degeneration.

In spite of the aforementioned evidence, activation of BMP pathways may also exert positive roles in motor neurons (Wang et al., 2007a; Chou et al., 2013; Kelly et al., 2013). For instance, long term differentiation (72 h) of NSC34 motor neuron cells in the presence of a Smad signaling inhibitor, show a reduction in the number of cells with long axons, which is counteracted by using BMP-4. Similarly, primary motor neuron cultures allowed to initiate axon growth show that BMP enhance, whereas the Smad inhibitor delay, motor axon advancement in culture (Kelly et al., 2013). These findings are in contrast to the role of BMP as an inhibitor of motor neuron differentiation. However, the delayed effect of the inhibitor (3 days of differentiation) on the neuritogenesis of NSC34 cells, and the experimental design in primary motor neurons to assess the effect on axon advancement, suggest that BMP signaling is required later on, once motor projections have already protruded from the cell soma (Kelly et al., 2013). Thus, a unifying hypothesis is that BMP signals could play dual effects on motor neurons: an early inhibitory role on morphological differentiation, and later, a positive role on axon elongation. Interestingly, we have shown that BMPRII is up-regulated throughout NSC34 cells differentiation and becomes accumulated in somas and growth cones. Remarkably, BMP-2 treatment, that inhibits neurite outgrowth, concomitantly up-regulates BMPRII (Benavente et al., 2012). Therefore, even though BMP-2 plays inhibitory roles on neurite outgrowth, it could also provide motor neuron cells with key proteins for their subsequent differentiation. Between these proteins, the BMPRII, which becomes localized in growth cones, could be involved in local signaling because of its ability to interact with cytoskeleton modulators (see before). Indeed, the BMP receptor type II is the most abundantly expressed BMP receptor in mouse spinal motor neurons (Wang et al., 2007a). Alternatively, the effect on motor neuron cells could be dependent on members of the BMP family or other related molecular players. In the ventral horn of normal adult rat spinal cord, motor neurons express BMP-2, BMP-4, noggin, BMPRIA, BMPRIB, and BMPRII (Miyagi et al., 2012). Therefore, Smad-dependent signaling via BMPRIs, local signaling via BMPRII, BMP-2 and BMP-4, exerting individual or combined effects, as well as noggin modulating BMP availability, could be relevant for normal motor neuron physiology such as embryonic synapse assembly, or during regeneration of neuronal connectivity.

## Concluding remarks

Besides the strong findings regarding the substantial role of Smad-dependent and independent signaling in invertebrate motor neurons, growing evidence reveals that BMPs also play important roles in the peripheral motor neuronal connectivity in vertebrate species. However, our understanding of the cellular effects of BMPs is still fragmented. Several studies suggest that the disturbance of BMP signaling is likely to be associated with a pathogenic status at the muscle fiber or the motor neurons.

Based in the literature and our studies, we propose a unifying hypothesis where BMP signal has dual effects on skeletal muscle and motor neurons. More related to the embryonic development of the neuromuscular connectivity, BMPs may have important roles in stimulating the amplification of muscle and motor neuron precursors, and simultaneously repressing precocious differentiation (Figure 2A). Indeed, there is convergence of neural and myogenic events within a neuromuscular synapse formation during vertebrate development (Mantilla and Sieck, 2008). Moreover, similar events could be recapitulated during axonal regeneration of peripheral nerves after injury. In fact, the recovery of contusion-induced spinal cord injury is accompanied by up-regulation of BMPRII, BMPRIA and Smad phosphorylation in spinal neurons (Matsuura et al., 2008). Likewise, Smad1 mRNA and protein levels are up-regulated in motor neurons after hypoglossal nerve injury in rats (Okuyama et al., 2007).

Afterwards, upon down-regulation of BMP signaling, the maturation of the neuromuscular synapse is reached. At this stage, BMP activation is triggered by the skeletal muscle and becomes restricted to the periphery of the postsynaptic domain, where it is modulated by the motor neuron-derived factor Agrin (Figure 2B). Based on these findings, it is tempting to speculate that these BMP signals could be related to axon elongation or presynaptic differentiation, as well as to NMJ formation, maturation or stability.

At present, it is still unclear how BMPs trigger different downstream signaling pathways. However, there are several possibilities including individual or mixed effects of: local concentrations of BMPs and receptors, expression of different BMPs and/or different combination of receptors expression. In this regard, it has been shown that BMP-7 specify the fate of dorsal interneurons at concentrations greater than two orders of magnitude than those required for induction of growth cone collapse (Perron and Dodd, 2011). In addition, BMPRI kinase activity that induces Smad-dependent signaling is required for cell specification but not for changes at the growth cone which require PI3K activity (Perron and Dodd, 2011). Other BMPs, including BMP-9, -4, -2, -5, 6, GDF-5 and -6 also have the ability to induce cell specification but only BMP-9, -4, -2 exhibit axon orientating activities (Perron and Dodd, 2012). Interestingly, immature mouse muscle fibers express BMP-6 whereas it is undetectable in muscle fibers or in post-synaptic domains (Wang et al., 2007a). In turn, BMP-4 is expressed in adult muscle cells and could mediate motor neuron-muscle interactions. Thus, either different levels or different BMPs expressed during the development of the muscle-motor neuron connectivity may generate different signaling events.

Further complexity arises from the expression of different receptors. For instance, it has been demonstrated a strong impact of BMP receptor utilization in conditions of BMPRII deletion (Yu et al., 2005). BMPRII expressing smooth muscle cells transduce BMP signals through BMPRII regardless of ActRIIa expression, indicating that the expression of BMPRII restrains BMP signaling via ActRIIa. BMPRII is the principal type II receptor for different classes of BMP ligands, transducing BMP-4/-2 signals through BMPRIa and also those from BMP-7/-6/-5 through either ActRIa or BMPRIa. In the absence of BMPRII, ActRIIa is the principal type II receptor for both classes of BMP ligands, transducing BMP-4/-2 signals through either ActRIa or BMPRIa and increasing BMP-7/-6/-5 signals potently through ActRIa (Yu et al., 2005). Therefore, changes in the global expression levels of BMPRII, or its local subcellular accumulation, could profoundly affect the gain or decrease of signaling activation by different BMP ligands.

Future work will provide a deeper understanding of the extracellular cues and cellular mechanisms mediated by BMPs underlying the interplay between muscle fibers and motor neurons in vertebrates. Experimental designs to establish the precise role of BMPs in muscle-motoneuronal communication must consider the evaluation of distinct cellular responses (Smad-dependent and independent), the spatial pattern of activation of specific signaling pathways in a time and concentration dependent manner, as well as if BMPs acts on cells at a distance from its source, which will imply to face these studies with morphogens criteria.

## Conflict of interest statement

The authors declare that the research was conducted in the absence of any commercial or financial relationships that could be construed as a potential conflict of interest.
